# Transceptors as a functional link of transporters and receptors

**DOI:** 10.15698/mic2017.03.560

**Published:** 2017-03-01

**Authors:** George Diallinas

**Affiliations:** 1 Department of Biology, National and Kapodistrian University of Athens, Panepistimioupolis 15784, Athens, Greece.

**Keywords:** Saccharomyces cerevisiae, fungi, signaling, PKA pathway, conformational change, endocytosis, evolution

## Abstract

Cells need to communicate with their environment in order to obtain nutrients, grow, divide and respond to signals related to adaptation in changing physiological conditions or stress. A very basic question in biology is how cells, especially of those organisms living in rapidly changing habitats, sense their environment. Apparently, this question is of particular importance to all free-living microorganisms. The critical role of receptors, transporters and channels, transmembrane proteins located in the plasma membrane of all types of cells, in signaling environmental changes is well established. A relative newcomer in environment sensing are the so called *transceptors*, membrane proteins that possess both solute transport and receptor-like signaling activities. Now, the transceptor concept is further enlarged to include micronutrient sensing via the iron and zinc high-affinity transporters of *Saccharomyces cerevisiae*. Interestingly, what seems to underline the transport and/or sensing function of receptors, transporters and transceptors is ligand-induced conformational alterations recognized by downstream intracellular effectors.

## THE ROLE OF PLASMA MEMBRANE PROTEINS IN DIRECT OR INDIRECT ENVIRONMENT SENSING

Three well-known types of molecular machines are employed for the sensing of the environment. Receptors, transporters and channels. All are polytopic transmembrane proteins, monomeric or oligomeric, located in the plasma membrane of all types of cells. Their importance is reflected by the fact that 10-15% of all genomes encode for such membrane proteins and by the numerous genetic diseases related to their malfunction. Receptors are proteins that interact with an external chemical ligand, which could be any simple molecule, including nutrients, ions, hormones or drugs and xenobiotics. Ligand binding induces a receptor conformational change, which acts as the primary molecular signal leading to two alternative downstream outcomes. In most cases, ligand-binding leads to the transduction of the receptor conformational change, *the signal*, into a cytoplasmic effector, usually an interacting protein at the inner side of the plasma membrane, which will further transduce the signal and modify gene expression accordingly. Well-studied cases of environmental sensing via typical receptors concern pH 1 or nutrient 2 sensing in fungi. Strikingly, the transmembrane receptors involved in nutrient sensing (e.g. Ssy1, Mep2, Snf3 and Rgt2) are structurally homologous to nutrient transporters. These receptors or sensors however have no transport activity and usually contain extended cytoplasmic domains not present in related transporters 3. In an alternative mechanism, ligand-binding elicits endocytic internalization of the receptor-ligand complex, and the ligand or the receptor, once in the cytoplasm, further transduces the signal on gene expression 4,5. In contrast to *bona fidae* receptors, transporters and channels mediate the uptake of solutes, metabolites, drugs or ions, which themselves can act as molecular signals, once intracellularly accumulated. A prominent case of transport-mediated signaling is described in filamentous ascomycetes, particularly in *Aspergillus nidulans*, where dormant conidiospores transferred to a fresh medium sense their new environment via transient transcriptional activation of most of their nutrient-related transporters and subsequently adapt their gene expression to the nutrients available 6. The primary signal that activates transporters in this case is not known, but is most probably related to conidiospore hydration, possibly via water channels. In fact water channels (aquaporins) seem to be involved in multiple signaling pathways, distinct from adaptation to nutrient availability, such as sporulation, cold tolerance, osmoregularity, modulation of cell surface properties and colony morphology, although the underlying molecular mechanisms in most cases remain unknown 7.

## THE DISCRETE APPEARANCE OF TRANSCEPTORS

In 1999, in the course of the 14^th^ Small Meeting of Yeast Transport and Energetics (SMYTE) held in Cordoba (Spain), Johan Thevelein presented evidence for a new type of sensing mechanism, operating however, through known transporters. In particular, he presented evidence that the general amino acid permease Gap1 of *Saccharomyces cerevisiae* is necessary for cAMP-independent activation of the PKA (Protein Kinase A) pathway under conditions of re-addition of amino acids to cells previously starved for amino acids 8. The PKA pathway plays a central role in the nutritional control of metabolism, stress resistance, cell cycle and growth, and is also regulated by other, cAMP-dependent, nutrient-sensing pathways which do not involve Gap1 9. Because Gap1 combined a transporter function with a PKA signaling, that is, a receptor function, it was named *transceptor* (***trans***port and rece***ptor***), and became the founder member of all transporters classified as transceptors today (see later). The basic original idea on the role of Gap1 in PKA activation was that signaling to the PKA pathway does not depend on the transport activity of Gap1. In other words, the role of Gap1 in activating PKA was independent from its role in amino acid transport. The evidence that Gap1 acts as a sensor was basically genetic. Strains carrying null or specific mutations in *GAP1* showed no PKA activation or distinct effects on transport and signaling, although these mutants could still transport high concentrations of amino acids that are Gap1 substrates via other specific permeases.

In the years to follow, Thevelein’s group has provided additional evidence supporting the concept of transceptors 10,11,12,13,14,15. The new evidence concerned mostly, but not only, Gap1. Several other high-affinity solute transporters have been shown to function as transceptors, namely those specific for phosphate (Pho84), ammonium (Mep2) and sulfate (Sul1 and Sul2). In the current issue of *Microbial Cell*, Thevelein and his colleagues further expand the family of transceptors by providing evidence that the iron (Ftr1) and zinc (Zrt1) high-affinity transporters can also signal PKA activation 16. Both metal transporters, similar to other transceptors are highly induced upon iron or zinc starvation and they are rapidly down-regulated by substrate-induced endocytosis. Thus, both macronutrients and micronutrients seem to elicit a common mechanism for signaling to the PKA pathway.

In summary, highlights of the evidence supporting the idea of transceptors include a) maintenance of signaling in specific transceptor transport-deficient versions, best exemplified by mutations in residues tentatively involved in proton-coupling in the Sul1 transceptor, b) loss of signaling in null mutants of transceptors despite accumulation of the relevant substrates by other transporters with similar specificity, c) identification of competitive and non-competitive inhibitors of Gap1-mediated transport, either with or without agonist action for signaling, including non-transported agonists, d) identification of analogues with distinct effects on Gap1 transport, transporter ubiquitination, endocytosis and signaling (transporter ubiquitination and endocytic turnover in response to substrate excess is a common phenomenon discussed below), and e) involvement of highly inducible scavenging transporters, as expected for proteins acting in signaling.

Overall, these results further support the hypothesis that different substrates, or substrate analogues, or ligands, bind to partially overlapping binding sites in the same general substrate-binding pocket of transceptors, triggering divergent conformations, resulting in distinct downstream processes, including ubiquitination, endocytic turnover or signaling 17. The somehow non-hierarchical effects caused by different substrates or ligands or the different transporter mutations, further suggest that specific conformational intermediates are differentially recognized by the specific effectors mediating the downstream processes.

## ACTIVITY-DEPENDENT CONFORMATIONAL CHANGES IN MEMBRANE PROTEINS: COMMON THEMES IN DOWNSTREAM OUTCOMES RELATED TO TRANSPORT AND SIGNALING

In my opinion, the idea of transceptors has gained unexpected conceptual support from studies addressing the phenomenon of activity-dependent transporter endocytosis. In fungi and mammalian cells many transporters are down-regulated in response to excess substrates by a mechanism involving ubiquitination, followed by immediate endocytic internalization and subsequent sorting in early endosomes and degradation in the vacuole/lysosome. Importantly, substrate-elicited transporter endocytosis, unlike endocytosis in response to other signals, such as stress or changes in N or C source supply, is activity-dependent and in most cases requires several transport cycles to be completed. This conclusion is mostly based on the observation that inactive transporters are, in most cases, not internalized in the presence of substrates. In line with the idea of the necessity of full transport cycles to be completed in order to allow endocytosis to occur, is the observation that substrate analogues that act as non-transported ligands do not elicit internalization. However, there are some exceptions to this rule. For example there are specific transporter mutants (e.g. in the UapA purine transporter in *A. nidulans* or in the Can1 arginine transporter in *S. cerevisiae*) that are active, or even hyperactive, but still not endocytosed, and some inactive mutants that are endocytosed 18,19. These observations supported the idea that substrates or ligands bind and elicit substrate/ligand-specific conformational changes critical for making a transporter vulnerable to ubiquitination and endocytosis 18,19,20. In fact, based on transporter structural modeling, mutagenesis and kinetic parameter analysis, current evidence further supports that substrate-elicited ubiquitination and subsequent endocytosis requires transition of a transporter to a cytoplasm-facing conformational state preceding substrate release into the cell 19,21.

**Figure 1:**
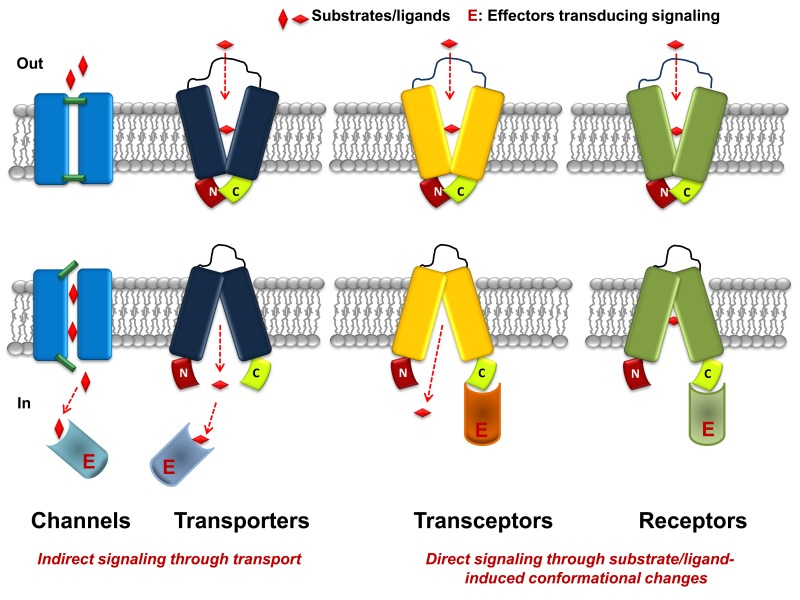
FIGURE 1: Highly speculative and simplified scheme comparing transporters, channels, transceptors and receptors. In channels ion transport (red vertical rhombs) is controlled by the simultaneous opening and closing of gates (green bars). Transporters, transceptors and receptors are shown to alternate from outward- to inward-facing conformations as a result of substrate or ligand (horizontal rhombs) binding. In the scheme the C-terminal domain of receptors and transceptors is arbitrary considered to be the domain recognized, specifically in its inward topology, by the downstream effector protein (E), which transduces the relative signal.

As proposed in signaling through transceptors, transporter activity is thus associated with a distinct mechanism, endocytosis, via specific conformational changes accompanying transport catalysis. In the case of transporters, conformational changes are ‘read’ by Rsp5/HulA/Nedd4 ubiquitin ligase adaptors (α-arrestins), which promote their internalization and turnover. In the case of transceptors, unknown effectors transduce conformational changes into the PKA signaling pathway. It thus seems that the cell has the capacity to sense subtle conformational nanochanges in transporters/transceptors, while they function at its plasma membrane. What is really fascinating about the discovery and the general concept of transceptors is that transceptors seem to provide a functional intermediate link in the apparent evolution of proper receptors from transporters 22 (**Figure 1**).

In the recent years, several research groups have provided evidence that transceptors are ubiquitously present in eukaryotes. In particular, nutrient transceptors for nitrate, ammonium, sulfate or nucleosides have been identified, at least tentatively, in filamentous fungi (*Neurospora crassa* and *Ustilago maydis*), plants (*Arabidopsis thaliana*), protozoa (*Leishmania mexicana*) and in human cells (see references in 16). However, classifying a transporter as a transceptor may lead to misinterpretation. Although uncoupling, genetically or biochemically, the transport and signaling functions might not always be feasible, technically or biologically, this still remains the best evidence for defining a transceptor. Thevelein’s group, very nicely developed genetic and biochemical assays to measure uptake and PKA signaling activation in parallel. Transport function was assayed directly in cells using radiolabelled substrates, while activation of PKA signaling is detected by measuring an increase in trehalase activity, a well-established PKA target 15. Two critical points to always be considered are whether a putative transceptor mutant has totally lost its transport activity, or that a substrate analogue used as a signaling ligand, is not transported in the cell.

In bacteria, yeast, filamentous fungi and other microorganisms, transport activity can be measured directly using radiolabeled substrates in living cells, a rigorous approach as the transporter activity is measured in its proper lipid environment. The only limiting factor in this approach might be the simultaneous expression of several transporters with similar or overlapping specificities and/or transport kinetics with the transporter studied. This apparent drawback is however overcome when a specific transporter can be studied in a genetically ‘clean’ background lacking, due to null mutations, transporters with similar specificities, as is the case in the model fungi *A. nidulans* and *S. cerevisiae*. Still however, absence of measurable transport activity of a radiolabelled substrate cannot guarantee that a very small amount of this substrate is not transported, at least when supplied at high concentrations. This is a purely technical problem, which mainly reflects cases where uptake assays are performed at relatively low concentrations of the radiolabeled substrate (usually at the μΜ range) and especially with low-affinity substrates. A different but complementary approach to direct uptake measurements approach is the use of growth tests in cases where the uptake of a substrate leads to a growth phenotype. For example, a strain of *S. cerevisiae *or* A. nidulans* expressing a transporter specific for adenine will grow well in growth media containing this purine as a sole nitrogen source, while it will also be sensitive to adenine toxic analogues, such 8-azaadenine. In contrast, inactivation of the same transporter will lead to absence of growth on adenine media and resistance to 8-azaadenine. Importantly, if similar growth tests are performed with a transporter over-expressed via a strong promoter, they can even allow the detection of transporters or transport mutants possessing very low activities. In respect to the identification of substrate analogues that only act as ligands, but still induce transporter-mediated signaling, rigorous evidence should be obtained that a substrate analogue is not accumulated, even at very low concentrations, in the cell, by another transporter, or even by the same transporter acting in signaling.

Several fascinating questions remain to be answered in respect to the mechanism of transceptor function. What distinguishes a transporter that acts solely in transport from a transporter that also acts as a receptor? Is this difference intrinsically based on structural determinants, or is it also a matter of transporter/transceptor localization in specific PM microdomains, and thus differentially accessible to downstream cellular effectors? Which *cis-*acting elements are necessary for signaling in different transceptors? Are the extended cytoplasmic terminal regions of some transceptors (e.g., Sul1/2) sufficient or critical for signaling, as it is in proper receptors (e.g. the Ssy1) or more complex synergistic intermolecular interactions determine signaling? And of course, which are the downstream effectors mediating transceptor signaling? Designing the proper genetic and unbiased screens in model fungi might lead to some answers to these questions.
